# Evidence for Cross-Species Transmission of Covert Mortality Nodavirus to New Host of *Mugilogobius abei*

**DOI:** 10.3389/fmicb.2018.01447

**Published:** 2018-07-09

**Authors:** Qing Li Zhang, Shuang Liu, Jun Li, Ting Ting Xu, Xiu Hua Wang, Guang Ming Fu, Xiao Ping Li, Song Wen Sang, Xiao Dong Bian, Jing Wei Hao

**Affiliations:** ^1^Qingdao Key Laboratory of Mariculture Epidemiology and Biosecurity, Key Laboratory of Maricultural Organism Disease Control, Ministry of Agriculture, Yellow Sea Fisheries Research Institute, Chinese Academy of Fishery Sciences, Qingdao, China; ^2^Laboratory for Marine Fisheries Science and Food Production Processes, Qingdao National Laboratory for Marine Science and Technology, Qingdao, China; ^3^School of Sciences and Medicine, Lake Superior State University, Sault Ste. Marie, MI, United States; ^4^Department of Pathology, The Affiliated Hospital of Qingdao University, Qingdao, China

**Keywords:** alphanodavirus, covert mortality nodavirus (CMNV), host jump, natural infection, *Mugilogobius abei*

## Abstract

Viral covert mortality disease (VCMD), caused by covert mortality nodavirus (CMNV), is a newly emerging disease affecting most cultured shrimp and other crustaceans, but not fish. However, we discovered for the first time that *Mugilogobius abei*, a common marine fish collecting from shrimp farming ponds and surrounding coastal waters in China, was tested to be CMNV positive based on reverse transcription loop-mediated isothermal amplification (RT-LAMP) assay. Further investigation based on the quantitative RT-LAMP assay indicated that 39% individuals of sampled *M. abei* were CMNV positive. Sequencing and alignment of sequences revealed that the partial RNA-dependent RNA polymerase gene of CMNV isolated from *M. abei* shared 98% homology with that from the original CMNV isolates. Histopathological analysis showed that CMNV infection in *M. abei* could induce extensive skeletal muscle necrosis, nervous tissue vacuolation in retina of eye and cerebellum of brain. Positive signals were verified in skeletal muscle, eye, brain and intestine by *in situ* hybridization (ISH) with CMNV probes. Under transmission electron microscope (TEM), CMNV particles were further visualized in the cytoplasm of neurogliocytes, granulocytes and myocytes in the CMNV positive samples diagnosed by ISH. All findings suggested that CMNV, a typical alphanodavirus originated from shrimp, could switch their hosts to fish by cross-species transmission. Meanwhile, the results reminded us to pay close attention to the high risk of CMNV to use fish as intermediate or new host as well as potentially spread or cause epidemic among cultured marine fish.

## Introduction

Nodamura virus (NoV) was the first identified species in the *Nodaviridae* and it was originally isolated from mosquitoes (*Culex tritaeniorhynchus*) sampled from the village of Nodamura near Tokyo of Japan in 1956 (Scherer and Hurlbut, 1967; Scherer et al., 1968; Tesh, 1980). Till now, over 25 members have been identified, which belong to two Genus, *Alphanodavirus* and *Betanodavirus* (Andrew et al., 2011). All alphanodaviruses were isolated in nature from insects and NoV is the type species of the *alphanodavirus genus* (Johnson et al., 2003). The alphanodaviruses can infect insects, whereas NoV is a unique one that can also lethally infect mammals (Scherer et al., 1968; Johnson et al., 2004). All betanodaviruses were isolated from larvae, juvenile or adult marine fish, in which they cause “viral nervous necrosis” or “viral encephalopathy and retinopathy” associated with abnormal behavior and high mortalities (Andrew et al., 2011). Previous reports confirmed that betanodaviruses are pathogenic to fish and can result in significant problems for the marine fish aquaculture industry (Chi et al., 2001; Ucko et al., 2004; Walker and Winton, 2010).

Covert mortality nodavirus (CMNV), a new member of *alphanodavirus*, was identified to be the infectious agent of the viral covert mortality disease (VCMD) of farming shrimp (Zhang et al., 2014, 2017b). Studies had confirmed that CMNV can infect major cultured crustaceans including *Penaeus vannamei*, *P. chinensis*, *Marsupenaeus japonicus*, *P. monodon*, and *Macrobrachium rosenbergii*; and caused serious loss of farming crustaceans in recent years (Pooljun et al., 2016; Thitamadee et al., 2016; Zhang et al., 2017b).

*Mugilogobius abei* is a commonly distributed marine fish species in shrimp farming ponds and surrounding coastal waters in China (Jordan and Snyder, 1901; Jin et al., 2014; Liu et al., 2015). Recently, a sample of *M. abei* from a shrimp farming ponds attacked by VCMD were tested to be CMNV positive by reverse transcription loop-mediated isothermal amplification (RT-LAMP) assay in our survey of CMNV natural host. CMNV positive of *M. abei* in level of nucleic acid detection clued that the *M. abei* might be infected naturally by CMNV. Up to now, alphanodaviruses have never been reported to be responsible to fish infection.

Following the clue of CMNV positive of the *M. abei* sample determined by RT-LAMP assay, we demonstrated the naturally infection of *M. abei* with CMNV based on a comprehensive investigation by using molecular histopathological, *in situ* hybridization (ISH) and transmission electron microscopic (TEM) assays in the present study. Our results provided the first evidence for supporting the host switching/extension of *Alphanodavirus* from shrimp to fish.

## Materials and Methods

### Sample Collection

Continuous monitoring of shrimp pathogens in a shrimp farming enterprise in Weifang City in China was executed by our laboratory from 2014 to 2018. The farming *Penaeus vannamei*, coexisting species of invertebrate and vertebrate in the ponds and the coastal water source of the enterprise, including *M. abei*, were free of CMNV before 2016. CMNV was introduced in several ponds of the enterprise through a starting offspring seeds infected by CMNV in May 2016. The farming *P*. *vannamei* in the ponds were attacked by VCMD from August 2016. So, *M. abei* samples (16∼21 mm in length), and live *Penaeus vannamei* samples (45∼98 mm in length), were collected from the VCMD attacked shrimp ponds and surrounding coastal water near the drainage channel of the enterprise. Nine *M. abei* individuals were sampled during 20th – 21st October 2016. Nine *M. abei* individuals and Five *P*. *vannamei* individuals were sampled in 15th August 2017 when the VCMD occurred again in the ponds. All the *M. abei* individuals looked normal in appearance, except some individuals showed abnormal swimming behaviors including vertically or horizontally spiraling movement.

Each individual sample was cut equally into two parts along the longitudinal axis and one part was preserved in 4% paraformaldehyde (4% PFA) solution (Sinopharm, Beijing, China). The other part was then divided into two parts and preserved respectively in RNAstore solution (Tiangen, Beijing, China) and 2.5% glutaraldehyde solution (Sinopharm, Beijing, China). These samples were applied for further analysis based on molecular, histopathological and electron microscopic approaches. The Ethics Committee of the Yellow Sea Fisheries Research Institute approved the use of animals and all procedure of operation in the study was complied with the national and institutional guidelines.

### RNA Purification

Total RNA was extracted from approximately 30 mg muscle tissues of *M. abei* by TRIzol Reagent (Invitrogen, Carlsbad, CA, United States) using TaKaRa MiniBEST Universal RNA Extraction Kit (Takara, Dalian, China) according to the manufacture’s instruction. The concentration and purity of extracted RNA was measured by Nanodrop 2000 (Thermo Scientific, Waltham, MA, United States).

### Detection of CMNV by Reverse Transcription Nested PCR and Sequencing

Total RNA samples of *M. abei* were submitted for diagnostic analysis of CMNV by reverse transcription nested PCR (RT-nPCR). Firstly, cDNA were synthesized from total RNA by using the SMART^®^ MMLV Reverse Transcriptase (TAKARA) with the primer of CMNV-7R1 according to the recommended procedures. The first step PCR were conducted by using CloneAmp HiFi PCR Premix (Takara) with the primers sets of CMNV-7F1/R1 and annealing at 50°C. The second step PCR was carried out according to our previous report with a minor modification (Zhang et al., 2014). The expected CMNV target fragments would be 619 bp amplicon and 413 bp amplicon after the first and second step of the PCR amplifications, respectively. The PCR products were purified by a PCR purification kit (Tiangen, Beijing, China) and then subjected for sequencing by the commercial sequencing company of Shanghai SANGAN Chemical Trading Co. Ltd.

### Phylogenetic Analysis

The target region (413 nt from nt no. 357 to 769) of CMNV RNA-dependent RNA polymerase (RdRp) gene of the second step PCR obtained in this study was aligned with the relevant sequences retrieved from the GeneBank database. Extra sequences were trimmed by comparing with the 413 nt CMNV RdRp gene sequence. All nucleotide sequences and deduced amino acid sequences were aligned using the ClustalW multiple alignment algorithm in the BioEdit 7.0. A phylogenetic tree was then constructed with bootstrap analysis (1000 replicates) using the software of MEGA 5.0 (Tamura et al., 2011).

### Quantitative Analysis of CMNV Infection

To quantify the CMNV copies in the *M. abei* samples, a quantitative reverse transcription loop-mediated isothermal amplification (qRT-LAMP) assay was applied to analyze the muscle tissues of *M. abei* according to the procedure previously described (Zhang et al., 2017a). A 10-fold serial dilution of the standard plasmid vector (pMD19-T) containing the target fragment from the CMNV RdRp gene was used as template to generate a standard curve for quantification. For the comparison, CMNV copies in the muscle tissues of *L. vannamei* were also quantified by using the same method.

### Histopathological Section

The samples were firstly incubated in 4% PFA fixative for 24 h, then changed to 70% ethanol, and followed by embedding the samples in paraffin blocks as the histological method reported by Bell and Lightner (1988). Triplicate of paraffin sections (3 μm) were prepared for histological and ISH analysis. Sections were stained with routine hematoxylin and eosin-phloxine (H&E) according to previously described procedures (Lightner, 1996). After checking the H&E stained sections, the corresponding unstained sections were subjected to CMNV ISH assay with digoxigenin (DIG)-labeled RNA probe.

### *In Situ* RNA Hybridization

A 244 bp DNA fragment of *RdRp* gene was amplified by using a set of primers with *Hind* III and *Pst* I recognition sites according to the previously reported protocol (Zhang et al., 2017b). The 244 bp DNA amplicons were digested to generate sticky ends of the dual-enzymes, and inserted into pBluescript II SK+ vector, followed by linearizing with *Hind* III and *Pst* I, respectively. The linearized vectors were used as template for transcription with T3 and T7 RNA polymerase for sense and antisense probes, respectively. In the procedure of transcription, DIG-NTP was added into the reaction to label the RNA probes. *In situ* RNA hybridization of each sample was conducted according to the protocols described previously (Piette et al., 2008; Chen et al., 2014). The sections post ISH were counterstained by using the Nuclear Fast Red solution as described elsewhere (Nuovo et al., 1999), and then visualized under the Nikon Eclipse E80i microscope (Nikon Co., Tokyo, Japan).

### Transmission Electron Microscopy

The samples preserved in 2.5% glutaraldehyde solution was subjected to further fixation with 1% osmium tetroxide, and dehydrated in a graded ethanol series, then embedded in Spurr’s resin and prepared ultrathin sections of 50 nm in thickness. The sections were stained with uranyl acetate and lead citrate in accordance with the previously reported protocols (Graham and Orenstein, 2007; Panphut et al., 2011). Ultrathin sections were prepared on collodion coated grids, and examined by using JEOL JEM-1200 electron microscope.

## Results

### Detection of CMNV by RT-nPCR

**FIGURE 1:**
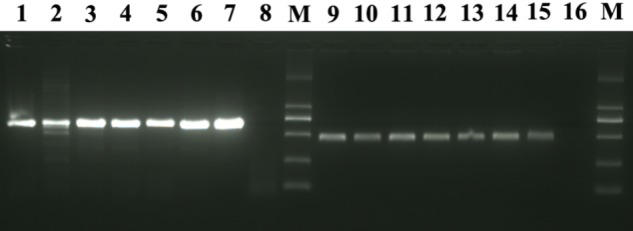
Electrophoretogram for detection of CMNV in *Mugilogobius abei* from shrimp farming ponds and coastal water by RT-nPCR. M: DL2000 molecular weight marker. Lane 1∼8 shows the result of the first step of RT-nPCR and Lane 9 ∼16 shows the result of the second step of RT-nPCR. Lane 1 and 9, lane 2 and 10, Lane 3 and 11 showed the amplification results of cDNAs from individuals collected from shrimp farming ponds. Lane 4 and 12, lane 5 and 13, Lane 6 and 14 showed the amplification results of cDNAs from individuals collected from coastal water near the ponds. Lane 7 and 15, lane 8 and 16 are the amplification results of cDNAs from the positive and negative control, respectively.

A total of six *M. abei* were diagnosed for CMNV by RT-nPCR. Three fish were collected from the shrimp farming ponds suffering VCMD and another three from the coastal water of surrounding area. The expected 619 bp amplicon of the first step PCR and the 413 bp amplicon of the second step PCR were amplified by using the cDNA templates from total RNAs of six *M. abei* samples despite of their sources (**Figure 1**). These findings indicated that all 6 *M. abei* samples were CMNV positive despite of their resources (**Figure 1**).

### Sequencing and Phylogenetic Analyses

**FIGURE 2:**
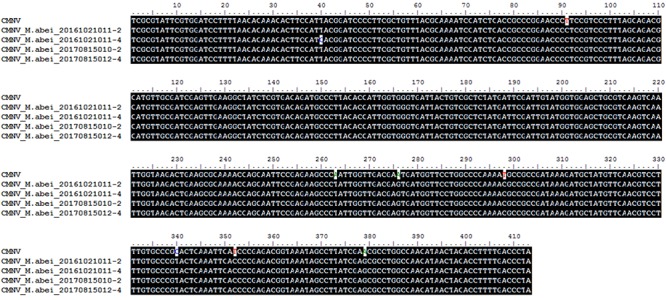
Alignments of the CMNV target gene from *Mugilogobius abei* with the corresponding gene (GenBank accession number KM112247) region of the original CMNV. The title of CMNV represented the RNA-dependent RNA polymerase (RdRp) gene from the original CMNV. The titles of CMNV_M. abei represented the RdRp gene from CMNV positive individuals of *M. abei*.

**FIGURE 3:**
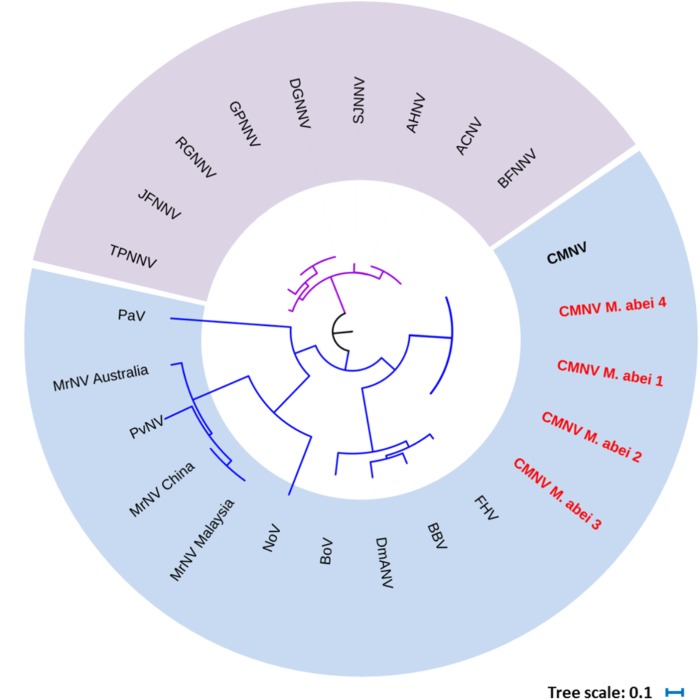
Phylogenetic tree based on the deduced amino acid sequences of RNA-dependent RNA polymerase (RdRp) gene from CMNV positive individuals of *Mugilogobius abei* and other nodaviruses (For virus abbreviations see **Table 1**). CMNV *M. abei* 1–2 were from individuals collected from shrimp farming ponds. CMNV *M. abei* 3–4 were from individuals collected from peripheral coastal water of ponds. The viral species of *Alphanodavirus* genus were indicated by baby blue background. The viral species of *Betanodaviruses* genus were indicated by lavender background. The tree was generated by the neighbor-joining method using the MEGA 5.0 program and the scale bar was 0.1.

BLAST search results indicated that the target gene fragments of all CMNV positive samples shared as high as 98% sequence similarity with the known CMNV target gene (KM112247) (**Figure 2**). Phylogenetic analysis showed that the CMNV target gene fragments from four different samples were clustered tightly into the branch of the original CMNV isolate, which demonstrated higher similarity with flock house virus (FHV), black beetle virus (BBV), Drosophila melanogaster American nodavirus (DmANV) and Boolarra virus (BoV) rather than *Macrobrachium rosenbergii* nodavirus (MrNV) and *Penaeus vannamei* nodavirus (PvNV) in the branch of *Alphanodavirus* (**Figure 3**). Meanwhile, all members from *Betanodavirus* were clustered into the other independent branch in the phylogenetic tree.

### Quantitation of CMNV Infection

Quantitation of viral copies in the muscle of eighteen *M. abei* samples and five *L. vannamei* samples were extrapolated based on the *Ct* value of the generated standard curve. The viral loads in the muscles of CMNV infected *M. abei* varied from 4.9 × 10^0^ to 3.5 × 10^4^ copies per mg tissues, which is lower than the viral loads in the muscles of *L. vannamei* (2.1 × 10^1^ to 8.3 × 10^5^). The quantitative RT-LAMP assay indicated that 39% individuals of sampled *M. abei* were CMNV positive (**Table 2**).

**Table 1:** Names and abbreviations for viral species of Nodaviridae.

Virus	Abbreviation	GenBank no.
Covert mortality nodavirus	CMNV	KM112247
Flock House virus	FHV	NP_689444
Black beetle virus	BBV	YP_053043
Macrobrachium rosenbergii nodavirus_China strain	MrNV China	AAQ54758
Macrobrachium rosenbergii nodavirus_Australia strain	MrNV Australia	AEY63648
Macrobrachium rosenbergii nodavirus_Malaysia strain	MrNV Malaysia	AEQ39078
Penaeus vannamei nodavirus	PvNV	YP_004207810
Drosophila melanogaster American nodavirus	DmANV	ACU32794
Nodamura virus	NoV	NP_077730
Boolarra virus	BoV	NP_689439
Pariacoto virus	PaV	NP_620109
Striped jack nervous necrosis virus	SJNNV	NP_599247
Tiger puffer nervous necrosis virus	TPNNV	YP_003288759
Atlantic halibut nodavirus	AHNV	AAY34458
Golden pompano nervous necrosis virus	GPNNV	ACX54065
Atlantic cod nodavirus	ACNV	ABR23192
Japanese flounder nervous necrosis virus	JFNNV	ACN58225
Dragon grouper nervous necrosis virus	DGNNV	AAU85148
Barfin flounder nervous necrosis virus	BFNNV	YP_003288756
Redspotted grouper nervous necrosis virus	RGNNV	ACX69744

*GenBank No. indicated the GenBank accession numbers of the amino acid sequence of RNA-dependent RNA polymerase used in this study*.

**Table 2:** Quantitation of viral copies in the muscle of *Mugilogobius abei* and *Penaeus vannamei*.

#	Sampling number	qRT-LAMP #copies	#	Sampling number	qRT-LAMP #copies
1	20161020001-1	None	14	20170815012-2	5.2 × 10^2^
2	20161020001-2	None	15	20170815012-3	None
3	20161020001-3	None	16	20170815012-4	4.9 × 10^0^
4	20161020005-2	1.7 × 10^2^	17	20170815012-5	None
5	20161020007-2	None	18	20170815012-6	None
6	20161021011-2	2.9 × 10^4^	***19***	*20170815001-1*	2.1 × 10^1^
7	20161021011-3	None	***20***	*20170815002-1*	*7.3 × 10^2^*
8	20161021011-4	6.1 × 10^2^	***21***	*20170815003-1*	*None*
9	20161021011-1	None	***22***	*20170815007-2*	*None*
10	20170815010-1	None	***23***	*20170815008-1*	8.3 × 10^5^
11	20170815010-2	8.7 × 10^2^	PC	20161103005-1	*2.9 × 10^4^*
12	20170815011-1	None	NC	20160804001-1	None
13	20170815012-1	3.5 × 10^4^	/		

*(1) #1-18 indicates the results for samples of *Mugilogobius abei*. #19-23 indicates the results for samples of shrimp *Litopenaeus vannamei*. (2) #copies indicate the copies in 1 mg tissues. (3) PC, positive control; NC, negative control*.

### Histopathological Changes Due to CMNV Infection

**FIGURE 4:**
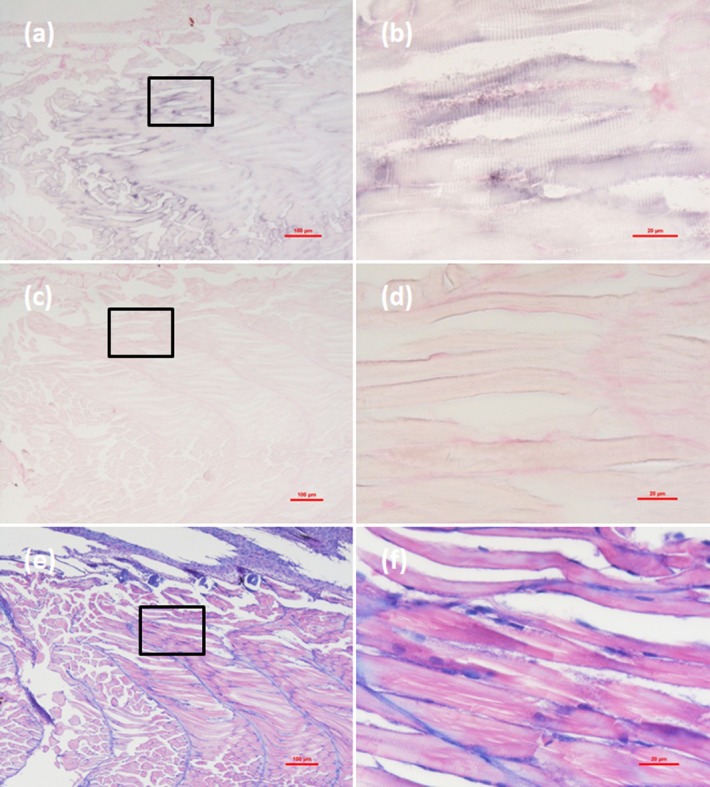
Micrographs of *in situ* hybridization (ISH) and H&E staining for necrotic skeletal muscle of the *Mugilogobius abei* naturally infected with CMNV. **(a)** Micrographs of ISH for skeletal muscle of CMNV-infected *M. abei* with the CMNV RNA probe. **(b)** Magnified micrograph of the area in the black frame in **(a)**. Note the intense hybridization signal at the dissolved myoneme of myocyte which demonstrated apparent lysis. **(c)** Micrographs of ISH for skeletal muscle of CMNV-infected *M. abei* without the CMNV RNA probe. **(d)** Magnified micrograph of the area in the black frame in **(c)**. **(e)** Micrographs of H&E staining for necrotic skeletal muscle of CMNV-infected *M. abei*. **(f)** Magnified micrograph of the area in the black frame in **(e)**. Note the haemocytic infiltration in fibromuscular stroma. Scale bars = **(a)** 100 μm, **(b)** 20 μm, **(c)** 100 μm, **(d)** 20 μm, **(e)** 100 μm, and **(f)** 20 μm.

**FIGURE 5:**
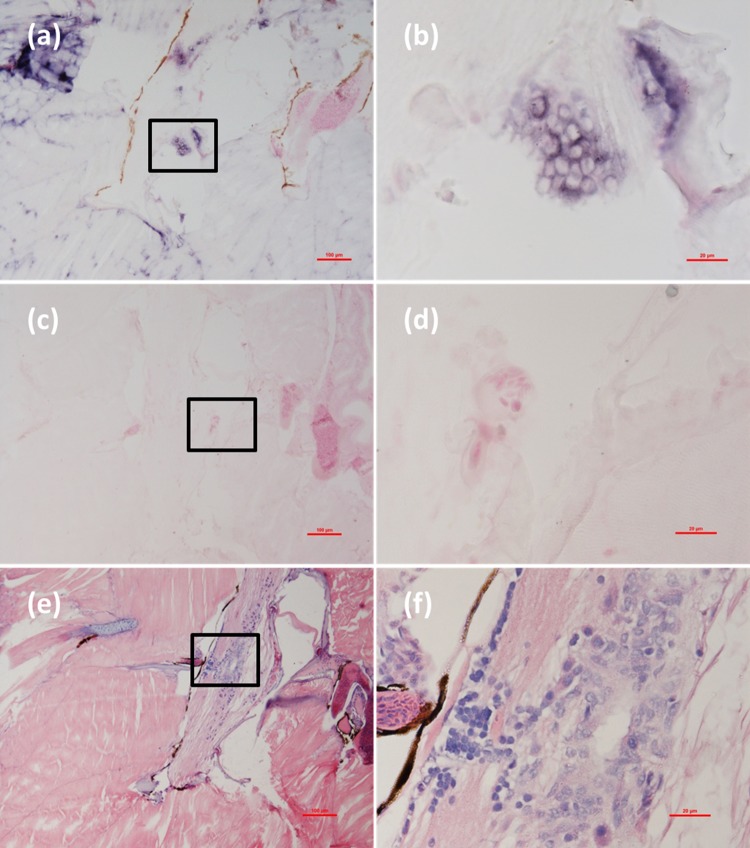
Micrographs of ISH and H&E staining for lesion of skeletal muscle with severe haemocytic infiltration of the *Mugilogobius abei* naturally infected with CMNV. **(a)** Micrographs of ISH for muscle lesion of CMNV-infected *M. abei* with the CMNV RNA probe. **(b)** Magnified micrograph of the area in the black frame in **(a)**. Note the intense hybridization signal in infiltrating hemocytes in the lysed muscle. **(c)** Micrographs of ISH for muscle lesion of CMNV-infected *M. abei* without the CMNV RNA probe. **(d)** Magnified micrograph of the area in the black frame in **(c)**. **(e)** Micrographs of H&E staining for muscle lesion of CMNV-infected *M. abei*. **(f)** Magnified micrograph of the area in the black frame in **(e)**. Scale bars = **(a)** 100 μm, **(b)** 20 μm, **(c)** 100 μm, **(d)** 20 μm, **(e)** 100 μm, and **(f)** 20 μm.

**FIGURE 6:**
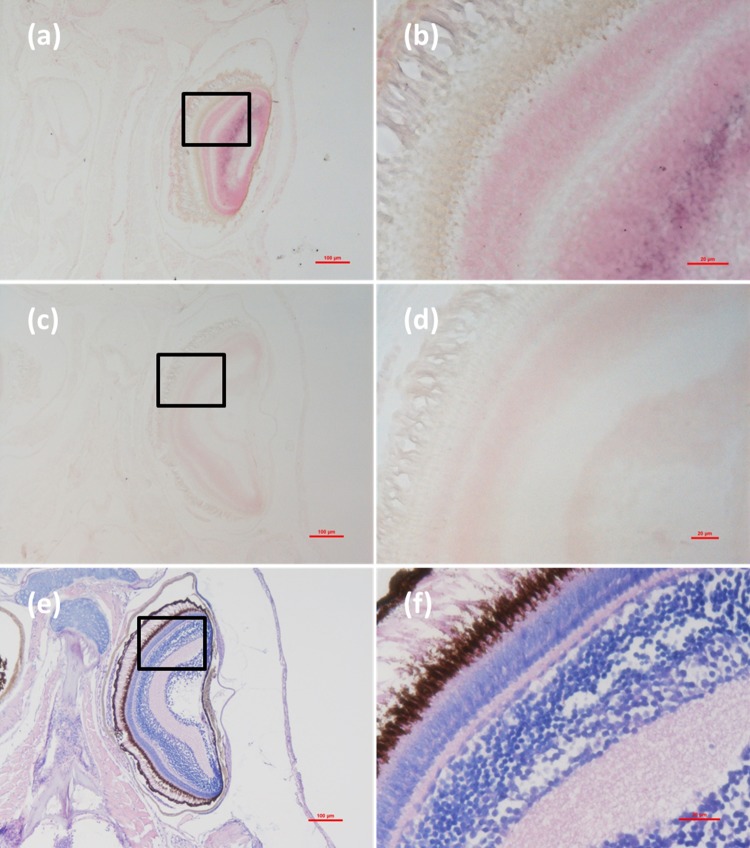
Micrographs of ISH and H&E staining for retina of the *Mugilogobius abei* naturally infected with CMNV. **(a)** Micrographs of ISH for retina of CMNV-infected *M. abei* with the CMNV RNA probe. **(b)** Magnified micrograph of the area in the black frame in **(a)**. Note the purple hybridization signal in the outer plexiform layer of the retina. **(c)** Micrographs of ISH for retina of CMNV-infected *M. abei* without the CMNV RNA probe. **(d)** Magnified micrograph of the area in the black frame in **(c)**. **(e)** Micrographs of H&E staining for the retina of eye from CMNV-infected *M. abei*. **(f)** Magnified micrograph of the area in the black frame in **(e)**. Scale bars = **(a)** 100 μm, **(b)** 20 μm, **(c)** 100 μm, **(d)** 20 μm, **(e)** 100 μm, and **(f)** 20 μm.

**FIGURE 7:**
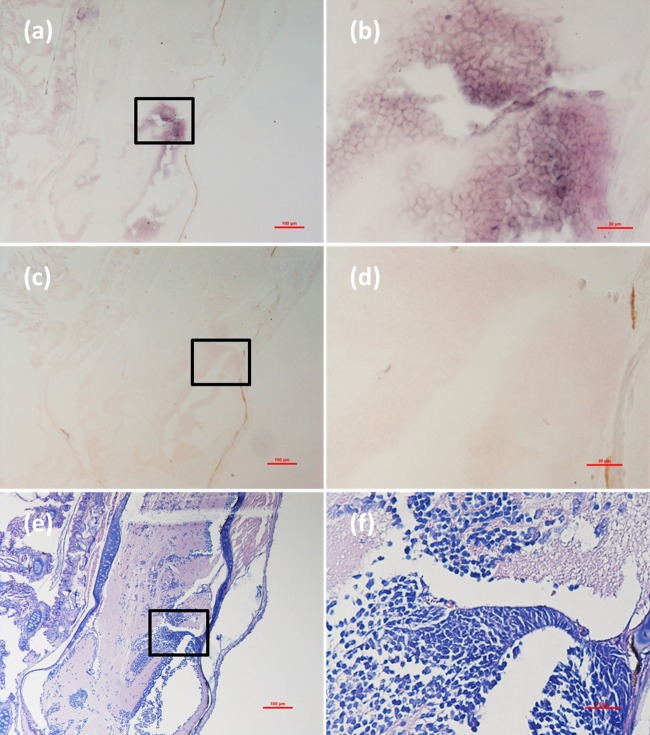
Micrographs of ISH and H&E staining for cerebellum of the *Mugilogobius abei* naturally infected with CMNV. **(a)** Micrographs of ISH for cerebellum of CMNV-infected *M. abei* with the CMNV RNA probe. **(b)** Magnified micrograph of the area in the black frame in **(a)**. Note the purple hybridization signal in the granule cell layer of the cerebellum. **(c)** Micrographs of ISH for cerebellum of CMNV-infected *M. abei* without the CMNV RNA probe. **(d)** Magnified micrograph of the area in the black frame in **(c)**. **(e)** Micrographs of H&E staining for the cerebellum from CMNV-infected *M. abei*. **(f)** Magnified micrograph of the area in the black frame in **(e)**. Scale bars = **(a)** 100 μm, **(b)** 20 μm, **(c)** 100 μm, **(d)** 20 μm, **(e)** 100 μm, and **(f)** 20 μm.

Histological examination revealed obvious histopathological alteration and lesions in the skeletal muscle, retina and brain of the *M. abei* samples. Extensive muscular lysis, myonecrosis (**Figures 4e,f**), and haemocytic infiltration in fibromuscular stroma (**Figures 5e,f**) could be observed from the examinated samples. Meanwhile, moderate vacuolation was noticed in the retina of eye and cerebellum of *M. abei* (**Figures 6e–f**). Haemocytic infiltration could also be observed among enterocytes of intestine folds (**Figures 7e–f**).

### Detection of CMNV in *M. abei* by ISH

**FIGURE 8:**
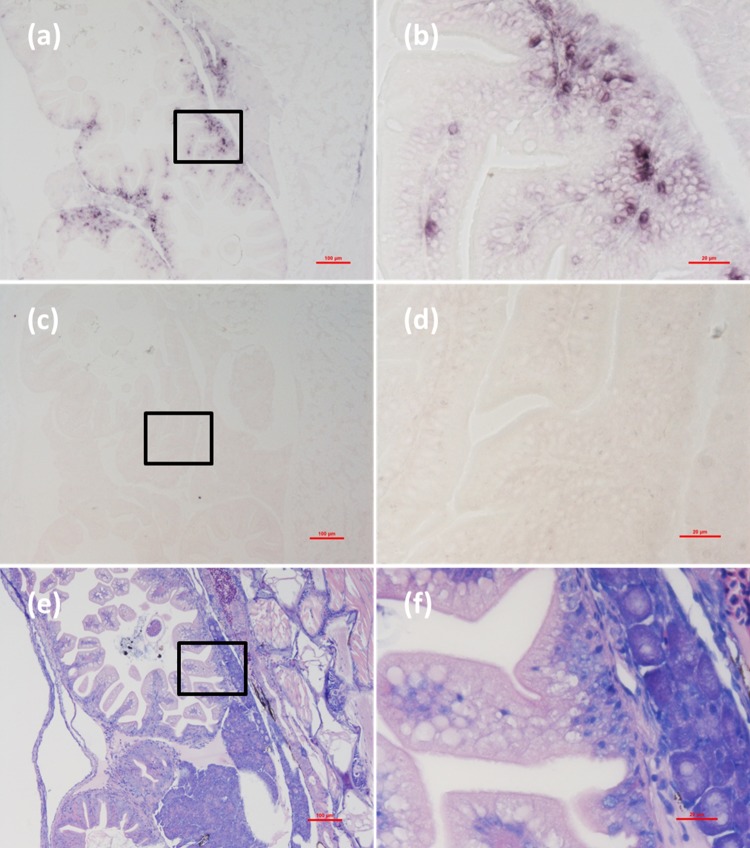
Micrographs of ISH and H&E staining for intestine of the *Mugilogobius abei* naturally infected with CMNV. **(a)** Micrographs of ISH for intestine of CMNV-infected *M. abei* with the CMNV RNA probe. **(b)** Magnified micrograph of the area in the black frame in **(a)**. Note the intense hybridization signal in the enterocytes of intestine. **(c)** Micrographs of ISH for intestine of CMNV-infected *M. abei* without the CMNV RNA probe. **(d)** Magnified micrograph of the area in the black frame in **(c)**. **(e)** Micrographs of H&E staining for the intestine from CMNV-infected *M. abei*. **(f)** Magnified micrograph of the area in the black frame in **(e)**. Scale bars = **(a)** 100 μm, **(b)** 20 μm, **(c)** 100 μm, **(d)** 20 μm, **(e)** 100 μm, and **(f)** 20 μm.

Micrographs of ISH for *M. abei* samples showed that the intense positive hybridization signals presented in the necrotic skeletal muscle (**Figure 4a**). Purple hybridization signal was clearly colocalized at the dissolved myoneme of myocyte which appeared apparent lysis at high magnification (**Figure 4b**). Infiltrating hemocytes in the lysed muscle showed intense hybridization signal of CMNV RNA probe as well (**Figures 5a,b**). Purple hybridization signal could be observed in retina outer plexiform layer of the eye, cerebellum granule cell layer of the brain, and enterocytes of intestine (**Figures 6**, **8a,b**), respectively. While, no positive hybridization signals appeared on the sections from both the same samples without CMNV RNA probe in the hybridization process (**Figures 4**–**8c–f**) and the CMNV negative sample determined by qRT-LAMP (see the Supplementary Data).

### Detection of CMNV in *M. abei* by TEM

**FIGURE 9:**
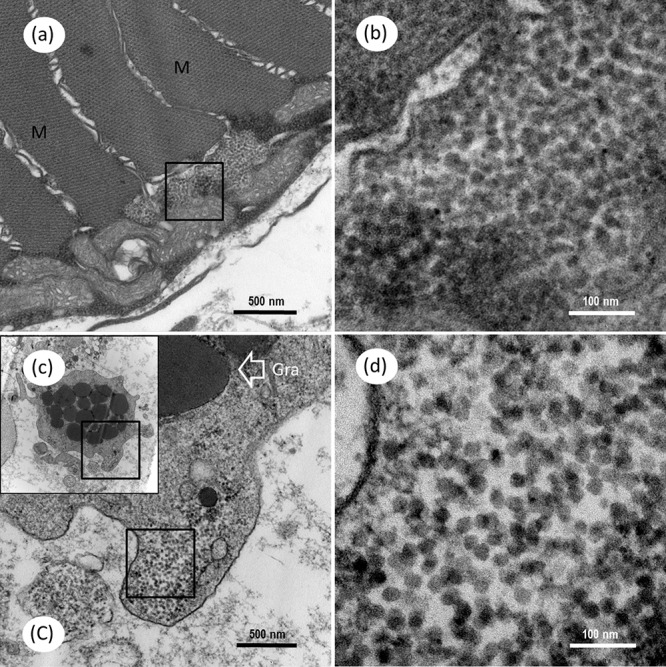
Transmission electron microscopic micrographs of ultrathin section for myocyte and granulocyte in skeletal muscle of *Mugilogobius abei.* **(a)** TEM of the myocyte of *M. abei*. **(b)** Magnified micrograph of the partial zone in the black frame in **(a)**. Note that the scattering distribution of CMNV-like particles near the mitochondria can be observed. **(c)** TEM of one granulocyte of *M. abei*. **(C)** Magnified micrograph of the partial zone of the granulocyte in the black frame in **(c)**. **(d)** Magnified micrograph of the partial zone of the granulocyte in the black frame in **(C)**. Note the scattering distribution of CMNV-like particles. Gra, granulocyte; M, Muscle; Scale bars = **(a)** 500 nm, **(b)** 100 nm, **(c)** 500 nm, and **(d)** 100 nm.

**FIGURE 10:**
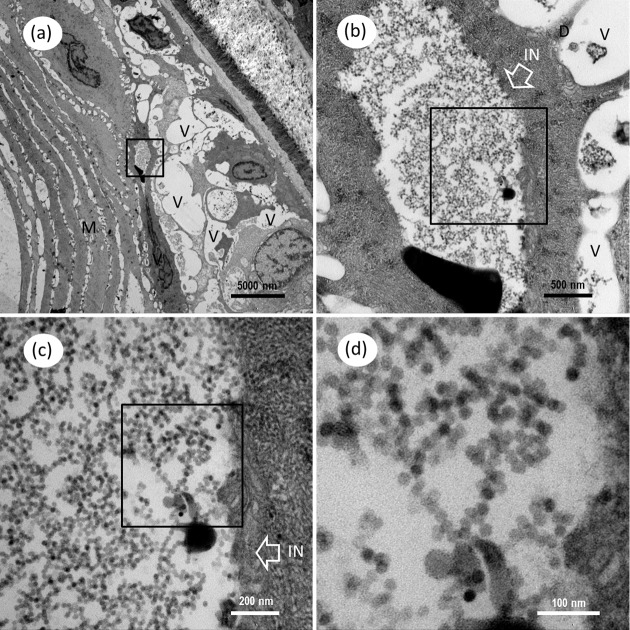
Transmission electron microscopic micrographs of ultrathin section for cerebellum of *Mugilogobius abei.* **(a)** TEM of the tissues of the cerebellum of *M. abei*. Conspicuous large vacuoles were noticed in the cerebellum of *M. abei*. **(b)** Magnified micrograph of the viral inclusion in the black frame in **(a)**. Viral inclusion was clear in the ultrathin section of the cerebellum. The white box arrow white arrows indicated the inclusion. **(c)** Magnified micrograph of the partial zone of viral inclusion in the black frame in **(b)**. **(d)** Magnified micrograph of the CMNV-like particles in the black frame in **(c)**. Note that the scattering distribution of CMNV-like particles can be observed. IN, Inclusion; M, Muscle; V, vacuole; N, nucleus; Scale bars = **(a)** 5000 nm, **(b)** 500 nm, **(c)** 200 nm, and **(d)** 100 nm.

Under the TEM, a group of unenveloped, virus-like particles were observed within the cytoplasm of myocyte, in which amounts of mitochondria surrounded the viral zone (**Figures 9a,b**). The ultrathin sections of skeletal muscle also revealed the presence of mass spherical CMNV-like particles with diameter about 24.7 ± 2.1 nm (*n* = 19) (**Figures 9c,C,d**) in the cytoplasm of granule cells. Conspicuous large vacuoles were noticed in the medulla oblongata of *M. abei* (**Figures 10a,b**) and numerous unenveloped CMNV-like particles could be found in the cytoplasm of adjacent neurogliocyte of the medulla oblongata (**Figures 10b–d**).

## Discussion

Alphanodaviruses were originally isolated from insects and their host range appears to be restricted to insects with the exception of the type species of Nodamura virus (NoV) and the Flock house virus (FHV) (Selling et al., 1990; Stock et al., 2009). NoV was a unique species among the alphanodaviruses and able to kill both insects and mammals (Bailey and Scott, 1973; Bailey et al., 1975). NoV had been reported to be infectious to pigs and transferable to sucking mice via a mosquito vector (Ball and Johnson, 1999). FHV, another insect originated alphanodavirus, could replicate in many species of plants including barley *Hordeum vulgare*, cowpea *Vigna sinensis*, chenopodium *Chenopodium hybridum*, tobacco *Nicotiana tabacum*, and *Nicotiana benthamiana* (Selling et al., 1990). It seemed that NoV and FHV possess the distinct capability for host switching. However, the host ranges of betanodavirus were different from alphanodaviruses and betanodavirus mainly infect larvae, juvenile or adult marine fish (Iwamoto et al., 2004; Furusawa et al., 2007; Souto et al., 2015). Up to now, except *Betanodavirus*, no any virus in the family of *Nordaviridae* has been reported to be capable to infect fish whether naturally or artificially.

**FIGURE 11:**
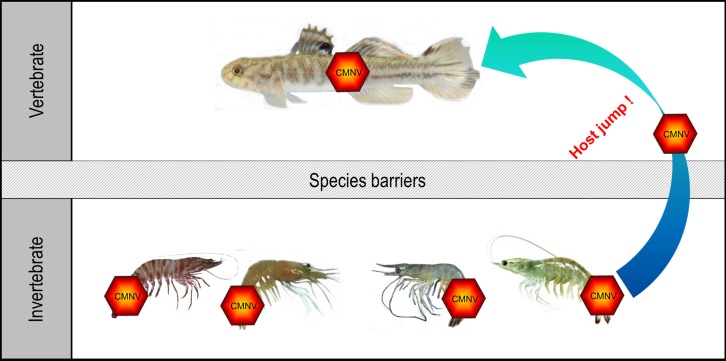
Host jump of CMNV from invertebrate to fish. The species of CMNV host in invertebrate included the *Penaeus japonicus*, *Exopalaemon carinicauda*, *Penaeus vannamei*, and *Penaeus chinensis*. This investigation revealed that CMNV, an emerging alphanodavirus originated from shrimp, possessed capability of crossing the species barriers and infecting *Mugilogobius abei*, a species of fish.

In the present study, we demonstrated for the first time that *M. abei*, a marine fish species, was naturally infected by CMNV, a member of alphanodavirus. Histopathological alteration and lesions, such as necrosis and heavy vacuolation, in the muscle, retina and cerebellum were very similar with that appeared in the target fishes infected by viral species of betanodavirus (Chi et al., 2001; Furusawa et al., 2007). Intense positive ISH signals in the necrotic myocyte indicated that CMNV could infect myocytes and caused muscular lysis in *M. abei*. The presence of CMNV particles in the cytoplasm of neurogliocytes, granule cells and skeletal muscle cells were further confirmed by TEM analysis. The result of natural infection of *M. abei* with CMNV provided the first clear evidence for that the viral member in alphanodaviruses can infect marine fish naturally (**Figure 11**).

Emerging viruses usually had RNA genome and as such were capable of rapid mutation and selection of new variants in the face of environmental changes in host numbers and available target species (Howard and Fletcher, 2012). A few viruses could be transmitted to completely new host species that they had never infected previously. Emerging viral diseases were often the product of a host shift, where a pathogen jumped from its original host into a novel species (Longdon et al., 2014). From the point of view of taxonomy, CMNV belonged to alphanodaviruses and the host range of viruses in this genus appeared to be restricted to insects. Nevertheless, CMNV was proved to be the pathogenic agent of VCMD, an emerging disease of farming shrimp, recently. This study supplied the novel evidence for CMNV host jump from shrimp to the co-existing marine fish in farming ponds and coastal water (**Figure 11**). These host transfers might involve either increased exposure to virus of the new host organism or the acquisition of viral variations that allow the virus to overcome species barriers to infection of the new hosts. Similar virus cases of host jump were reported occasionally (Parrish et al., 2008; Kreuder et al., 2015) and several typical documentations included that a plant virus switching hosts to infect a vertebrate (Gibbs and Weiller, 1999), H7N9 avian influenza starting to infect humans (Shi et al., 2014) and permanent host shift of rabies virus from Chiroptera to Carnivora (Ding et al., 2017).

*Mugilogobius abei* was a species of fish in the family *Gobiidae* and mainly distributes in fresh, brackish and marine water of the Indo-Pacific region (Larson, 2001; Huang et al., 2015). It was the keystone species of bait fish in the Bohai Sea, the Yellow Sea, and the East China Sea and played important roles in the food web of the coastal water communities in the Sea (Jin et al., 2014; Liu et al., 2015, 2016). In this study, a total of 18 individuals of *M. abei*, collected either from shrimp farming ponds or surrounding coastal waters, were analyzed and seven individuals were CMNV positive based on RT-LAMP. The highest viral load in the CMNV positive samples was up to 3.5 × 10^4^ viral copies per mg tissues. Natural infection of *M. abei* from coastal waters with CMNV, along with the fact of the high infection rate and infective dose of CMNV in *M. abei* samples, might alert CMNV as an emerging and significant hazards factor to the natural population of *M. abei* in coastal area. Recently, we identified CMNV from nearshore *Chaeturichthys hexanema*, another wild marine fish in the Yellow Sea, based on the RT-LAMP and RT-nPCR assay. All these results revealed the substantial risk of wide prevalence of CMNV in farmed and wild marine fish species.

## Conclusion

Natural infection of *M. abei* with CMNV demonstrated by present study supplied the first evidence for alphanodavirus infecting fish. Our findings suggested that CMNV possessed the distinct capability of host jump and it could infect marine fish, *M. abei*, which was a comman species in shrimp farming ponds and a dominant species in coastal water in China. The result prompted that CMNV would be an important and emerging ecological risk for its infectious ability and pathogenicity to marine fish.

## Author Contributions

QZ and SL designed, executed the experiments and analyzed the samples. SL conducted the *in situ* RNA hybridization assay. XL and TX did molecular and biological analysis. QZ conducted the TEM assay. XW and TX contributed to sampling. XB helped to identify the species of the fish samples. GF, SL, and JH prepared the histological sections. SS did the sequencing work. QZ and JL wrote the manuscript. All authors interpreted the data, critically revised the manuscript for important intellectual contents and approved the final version.

## Conflict of Interest Statement

The authors declare that the research was conducted in the absence of any commercial or financial relationships that could be construed as a potential conflict of interest.
