# Supplementary material for: Evidence for Cross-Species Transmission of Covert Mortality Nodavirus to New Host of Mugilogobius abei
Source: Front Microbiol. 2018 Jul 9;9:1447. doi: 10.3389/fmicb.2018.01447 (PMC6046410; doi:10.3389/fmicb.2018.01447)
Supplement: Supplementary file 1 [file Data_Sheet_1.docx]

**Supplementary Data:** No positive hybridization signals appeared on the sections from the CMNV negative sample determined by qRT-LAMP.


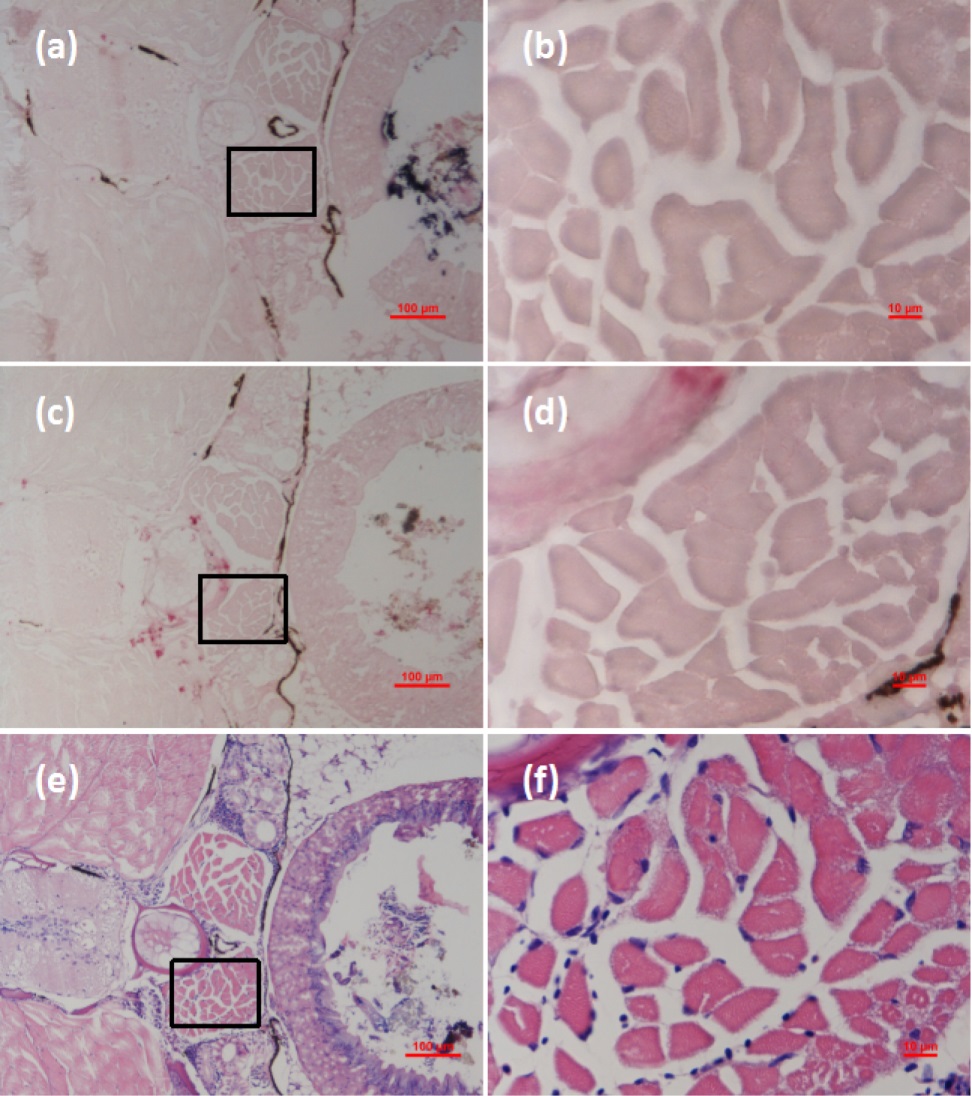


**Figure caption:** Micrographs of *in situ* hybridization and H&E staining for skeletal muscle of the CMNV negative *Mugilogobius abei* sample determined by qRT-LAMP. (a) Micrographs of ISH for muscle of CMNV negative *M. abei* with the CMNV RNA probe. (b) Magnified micrograph of the area in the black frame in (a). Note that there was no hybridization signal of CMNV probe in the muscle. (c) Micrographs of ISH for muscle of CMNV negative *M. abei* without the CMNV RNA probe. (d) Magnified micrograph of the area in the black frame in (c). (e) Micrographs of H&E staining for muscle of CMNV negative *M. abei*. (f) Magnified micrograph of the area in the black frame in (e). Scale bars = (a) 100 μm, (b) 20 μm, (c) 100 μm, (d) 20 μm, (e) 100 μm, (f) 20 μm.
